# Correction: Complement activation by gold nanoparticles passivated with polyelectrolyte ligands

**DOI:** 10.1039/c8ra90017e

**Published:** 2018-02-21

**Authors:** Quang Huy Quach, James Chen Yong Kah

**Affiliations:** Department of Biomedical Engineering, Faculty of Engineering, National University of Singapore Singapore 117583 Singapore; NUS Graduate School for Integrative Science and Engineering, National University of Singapore Singapore biekahj@nus.edu.sg

## Abstract

Correction for ‘Complement activation by gold nanoparticles passivated with polyelectrolyte ligands’ by Quang Huy Quach *et al.*, *RSC Adv.*, 2018, **8**, 6616–6619.

The authors regret that James Chen Yong Kah’s name was presented incorrectly in the original article. The correct spelling of all author names is presented above.

The Royal Society of Chemistry apologises for these errors and any consequent inconvenience to authors and readers.

## Supplementary Material

